# Blood Bacterial 16S rRNA Gene Alterations in Women With Polycystic Ovary Syndrome

**DOI:** 10.3389/fendo.2022.814520

**Published:** 2022-02-24

**Authors:** Qing Wang, Qi Wang, Lanbo Zhao, Yadi Bin, Li Wang, Lei Wang, Kailu Zhang, Qiling Li

**Affiliations:** Obstetrics and Gynecology Department, The First Affiliated Hospital of Xi’an Jiaotong University, Xi’an, China

**Keywords:** PCOS, blood, microbiome, 16s rRNA, sequencing

## Abstract

**Background:**

Evidence proved the association between gut microbiome dysbiosis and polycystic ovary syndrome (PCOS) in metabolic disorder, decreased fertility, and hyperandrogenism. However, alterations in blood microbiome of PCOS remained unknown.

**Objective:**

This study aims to measure the blood microbiome profile of PCOS patients compared with healthy controls by 16S rRNA sequencing and to investigate its association with PCOS.

**Methods:**

In this case–control study, bacterial DNA in blood of 24 PCOS patients and 24 healthy controls was investigated by 16S rRNA gene sequencing using the MiSeq technology. Alpha and beta diversity were used to analyze within-sample biodiversity and similarity of one group to another, respectively. Linear discriminant analysis effect size (LEfSe) was calculated to determine biomarkers between groups. Kyoto Encyclopedia of Genes and Genomes (KEGG) functional prediction was performed at genera level.

**Result:**

Alpha diversity of blood microbiome decreased significantly in women with PCOS, and beta diversity analysis demonstrated a major separation between the two groups. In the PCOS group, the relative abundance of *Proteobacteria*, *Firmicutes*, and *Bacteroidetes* decreased significantly, while *Actinobacteria* increased significantly. Cladogram demonstrated the microbiome differences between the two groups at various phylogenic levels. Meanwhile, linear discriminant analysis (LDA) presented significant decreases in *Burkholderiaceae*, *Lachnospiraceae*, *Bacteroidaceae*, *Ruminococcaceae*, and *S24-7* and significant increases in *Nocardioidaceae* and *Oxalobacteraceae* of the PCOS group. KEGG pathway analysis at genera level suggested that 14 pathways had significant differences between the two groups.

**Conclusion:**

Our findings demonstrated that blood microbiome had a significantly lower alpha diversity, different beta diversity, and significant taxonomic variations in PCOS patients compared with healthy controls.

## Introduction

Polycystic ovary syndrome (PCOS) is a common but complex endocrine and metabolic disease, which may increase the risk of infertility, type 2 diabetes, insulin resistance, and obesity (1, 2). Many factors, such as heredity and lifestyle, are related to this disease. However, the exact reason for all these metabolic and biochemical disorders is still unclear (3).

Microbiome was the hotspot in many studies. It was reported that the microbiome played roles in regulating immune and digestive systems, and it was associated with metabolic diseases (4). Many scholars had been researching on the association between gut microbiome and diseases. However, the exact mechanism of how gut microbiome impacted on the body remained a mystery. Whether gut microbiome just rooted in the gut system modulating remotely or intruded into targeted tissue through the circulatory system, or merely infiltrated adjacently, still puzzled the researchers. Hence, some researchers shifted focus on the circulatory microbiome.

More and more evidence proved the existence of blood bacteria in healthy humans, and numerous studies focused on the potential connections between circulating microbiome variation and the pathogenesis of non-infectious diseases (5–8). Lelouvier et al. revealed that alterations of blood microbiome were related to liver fibrosis in obese patients (9), which was confirmed by another study in which the blood samples were obtained from hepatic outflow vessels, central and peripheral veins, and the portal veins in patients (10). *Sediminibacterium* in blood increased the risk of type 2 diabetes mellitus [adjusted odd ratio (OR), 14.098; 95% confidence intervals (CIs), 1.358–146.330], while *Bacteroides* in blood decreased this risk significantly (adjusted OR, 0.367; 95% CI, 0.151–0.894) (11). Higher levels of blood bacterial diversity were detected in patients with cirrhosis compared to healthy controls (12). Serum microbiome composition in gastric cancer group was significantly different from that of atypical hyperplasia, chronic gastritis, and healthy control groups (13). Contrasted with healthy controls, patients with chronic kidney disease had lower alpha diversity and significant taxonomic variations in the blood microbiome detected by 16S targeted metagenomic sequencing (14).

As for the research about PCOS and microbiome, recent studies confirmed that the gut microbiome dysbiosis was associated with PCOS in metabolic disorders, sterility, and hyperandrogenism (15–17). Qi et al. detected increased abundance of *Bacteroides vulgatus* in the gut microbiota of PCOS patients and proved its association with metabolic pathway of gut microbiota–bile acid–interleukin-22 (18). Decreased alpha diversity (species richness and phylogenetic diversity) and changed beta diversity of gut bacteria composition were observed in patients with PCOS (16, 17, 19). Lindheim et al. revealed a reduced salivary *Actinobacteria* abundance in PCOS patients, and they reported that saliva microbiome was also associated with reproductive, metabolic, and inflammatory parameters (20).

However, no study about blood microbiome in women with PCOS had been reported. Here, we aimed to measure the blood microbiome profile of PCOS patients compared with healthy controls by 16S rRNA sequencing and to investigate its association with PCOS.

## Methods

### Patients

A total of 24 women in the PCOS group and 24 participants in the healthy control group were enrolled from the First Affiliated Hospital of Xi’an Jiaotong University. Inclusion criterion of the PCOS group was newly diagnosed PCOS patients following the Rotterdam criteria (with at least two of the three characteristics of clinical and/or biochemical hyperandrogenism, ovulatory dysfunction, and polycystic ovarian morphology). Inclusion criterion of the healthy control group was women without history of PCOS. Exclusion criteria for all participants were the following: (1) adrenal disorder; (2) pregnancy; (3) Cushing’s syndrome; (4) androgen-secreting tumors; (5) administration of oral contraception; (6) fever, or infectious disease, or antibiotic usage within 3 months; and (7) chronic diseases like chronic kidney disease, liver disease, rheumatoid arthritis and so on. The study was approved by the Institutional Review Board of the First Affiliated Hospital of Xi’an Jiaotong University (IRB No. XJTU1AF2018LSK139) and was registered in the Chinese Clinical Trial Registry (ChiCTR-TRC-1800020018). Informed consent was obtained from all individuals.

### DNA Extraction and 16s rRNA Sequencing

For each participant, fasting blood was collected in an ethylenediaminetetraacetic acid (EDTA) tube and stored at −80°C. DNA was extracted using the Mag-Bind^®^ Pathogen DNA 96 Kit (Omega Biotek, Norcross, GA, USA). After quality and concentration tests, the V3–V4 hypervariable regions of bacterial 16S ribosomal gene were quantified by quantitative PCR (qPCR) and sequenced by the MiSeq technology (Illumina, San Diego, CA, USA), as described previously (21). Briefly, 16s rRNA was processed with hybrid *de novo*, a secondary processing pipeline (22), which took paired reads as input; a *de novo* operational taxonomic units (OTUs) picking and output OTU abundance and annotations as the Biological Observation Matrix (BIOM) format were performed. Sequences were clustered into OTU at a threshold of 97% sequence similarity before taxonomic assignment.

### LEfSe Analysis

Linear discriminant analysis effect size (LEfSe) was calculated to determine biomarkers between groups with an absolute linear discriminant analysis (LDA) score >3.0 as cutoff value. The relative abundance of specific species was logarithmically transformed and shown by bar diagrams. Differences between groups were tested by the Mann–Whitney method.

### Kyoto Encyclopedia of Genes and Genomes

With the BIOM file, Kyoto Encyclopedia of Genes and Genomes (KEGG) orthologs functional predictions were performed using Phylogenetic Investigation of Communities by Reconstruction of Unobserved States (PICRUSt) and visualized by Statistical Analysis of Metagenomic Profiles (STAMP) (23), where the genus was the profile level and an effective size of 0.1 was set as the cutoff value.

### Statistical Analysis

Clinical characteristics between PCOS and control groups were compared by *t*-test. Normal distribution of bacteria was calculated by Kolmogorov–Smirnov test. The Mann–Whitney U-test was used to evaluate bacterial composition and abundance between the two groups from different levels (phyla, order, class, and family). Alpha diversity of each sample was shown by Chao1 index, Faith’s phylogenetic diversity, and Observed_OTUs. Beta diversity between pairs of individual samples was measured by weighted and unweighted UniFrac analysis using the Quantitative Insights into Microbial Ecology (QIIME version:1.9.1). LEfSe algorithm was used to compute OTU differences between the two groups, and LDA value >3.0 was considered to be rich significantly.

## Results

### Baseline Characteristics

Baseline characteristics of 48 participants are shown in **Table 1**. Ages were ranged between 18 and 39 years old and 23 and 34 years old in the PCOS and control groups, respectively. Body mass index (BMI) in the PCOS group was significantly higher than that of the control group (25.90 ± 4.68 vs. 21.39 ± 3.00, *p* = 0.017). Among the 24 patients with PCOS, there were 23 complaining of oligomenorrhea, 16 with polycystic ovarian morphology diagnosed by ultrasound examination, and 13 with clinical symptom of hyperandrogenism. Women in both groups had no significant difference in leukocyte, neutrocyte, neutrophil percentage, hemoglobin, thyroid-stimulating hormone, or fasting plasma glucose.

**Table 1:** Baseline characteristics of participants.

	PCOS (n = 24)	CONTROL (n = 24)	*p*
Age (year)	24.92 ± 5.13	28.20 ± 3.00	0.037
BMI (kg/m^2^)	25.90 ± 4.68	21.39 ± 3.00	0.017
Oligomenorrhea	23	0	–
PCOM	16	0	–
Hyperandrogenism	13	1	–
Leukocyte (10^9^/L)	6.23 ± 1.52	5.96 ± 1.76	0.638
Neutrocyte (10^9^/L)	3.50 ± 1.25	3.90 ± 1.53	0.885
Neutrophil percentage (%)	55.54 ± 8.70	64.81 ± 7.94	0.891
Hemoglobin (g/L)	137.44 ± 8.87	134.00 ± 14.99	0.159
TSH (uIU/ml)	2.38 ± 1.58	2.34 ± 1.54	0.629
FPG (mmol/L)	5.03 ± 2.49	4.62 ± 0.47	0.169

BMI, body mass index; PCOM, polycystic ovarian morphology; TSH, thyroid-stimulating hormone; FPG, fasting plasma glucose.

### Diversity of the Blood Microbiome

**Figure 1:**
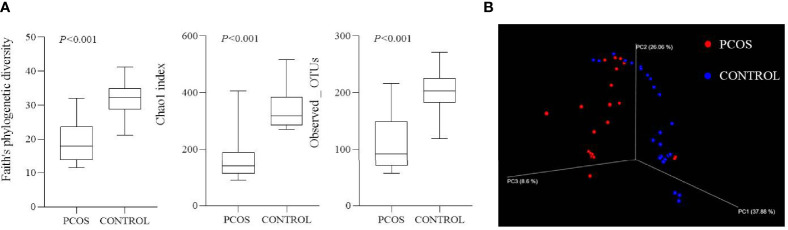
Alpha and beta diversity of two groups. **(A)** Alpha diversity of two groups using Faith’s phylogenetic diversity, Chao1 index, and Observed_OTUs (all *p* < 0.001). **(B)** Beta diversity shown by principal coordinates analysis using weighted UniFrac analysis.

Patients in the PCOS group displayed a significant decrease in alpha diversity, a lower within-sample biodiversity, compared with control participants using Faith’s phylogenetic diversity (19.04 ± 6.08 vs. 32.05 ± 4.77), Chao1 index (160.80 ± 76.66 vs. 337.87 ± 64.98), and Observed_OTUs (105.47 ± 44.68 vs. 202.91 ± 37.27) (all *p* < 0.001, **Figure 1A**). A major separation in beta diversity between groups was observed from principal coordinates analysis by weighted UniFrac analysis (**Figure 1B**).

### Circulating Microbiome Community Structure

16s rRNA sequences were obtained from 24 PCOS and 24 healthy control participants. After trimming and quality evaluation, 1,552,041 reads were counted in total (32,334.2 ± 25,845.6 per sample). A total of 38 phyla, 107 classes, 212 orders, and 354 families were identified in blood microbiome of the two groups.

**Figure 2:**
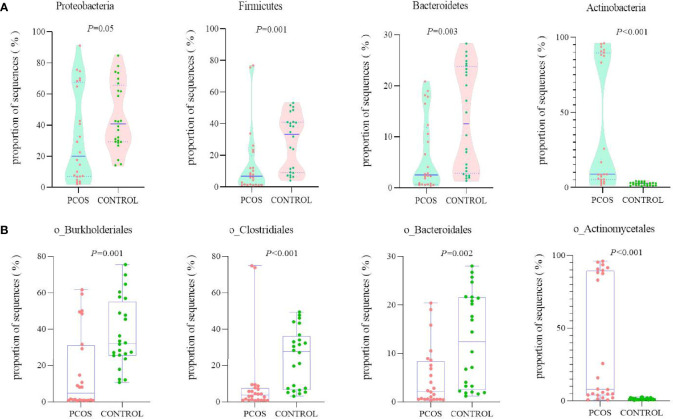
Comparison of the relative abundance of the top 4 richest bacteria between PCOS and control groups at two levels. **(A)** Phylum; **(B)** order.

The total sequencing proportion of primary blood bacteria from different levels is shown in **Supplementary Figure S1**. At the phylum level, blood microbiome was predominately composed of *Proteobacteria* (38.70%), followed by *Firmicutes* (21.30%), *Actinobacteria* (20.60%), and *Bacteroidetes* (10.00%). These four comprised approximately 90.60% of all phyla. The PCOS group revealed significantly lower proportions in sequences of *Proteobacteria*, *Firmicutes* and *Bacteroidetes* and a significantly richer abundance in *Actinobacteria* compared with that of the control group (**Figure 2A**). Same differences were observed when tracing main branches of these four phyla into order level, such as *Burkholderiales*, *Clostridiales*, *Bacteroidales*, and *Actinomycetales* (**Figure 2B**). Further analysis was performed on the 17 most abundant bacteria at class level between the two groups, and 12 of them had significant differences. The PCOS group exhibited significant increased proportions in *Intrasporangiaceae*, *Nocardioidaceae*, *Bacteroidaceae*, *Comamonadaceae*, *Oxalobacteraceae*, and *Moraxellaceae*, whereas significant decreases in *S24-7*, *Lactobacillaceae*, *Lachnospiraceae*, *Ruminococcaceae*, *Veillonellaceae*, and *Burkholderiaceae* than that of the control group (**Supplementary Figure S2**).

### Taxonomic Signature Analysis

**Figure 3:**
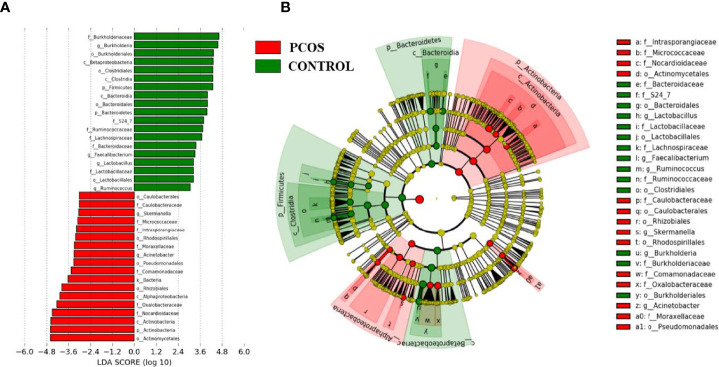
Linear discriminant analysis effect size (LEfSe) analysis of the two groups. **(A)** Linear discriminant analysis (LDA) represented statistical and biological differences between the two groups (LDA > 3.0, *p* < 0.05). **(B)** Cladogram demonstrated microbiome differences of the two groups at various phylogenic levels.

After taxonomic profile analysis of each participant, LEfSe demonstrated significant differences at some taxonomic levels between the two groups with threshold score of LDA >3.0 (**Figure 3**). Cladogram showed microbiome differences in the two groups at various phylogenic levels. Compared with the control group, the PCOS group presented significant increases in *Nocardioidaceae* and *Oxalobacteraceae*, while it presented significant decreases in *Burkholderiaceae*, *Lachnospiraceae*, *Bacteroidaceae*, *Ruminococcaceae*, and *S24-7*.

### Functional Prediction of Blood Bacteria

**Figure 4:**
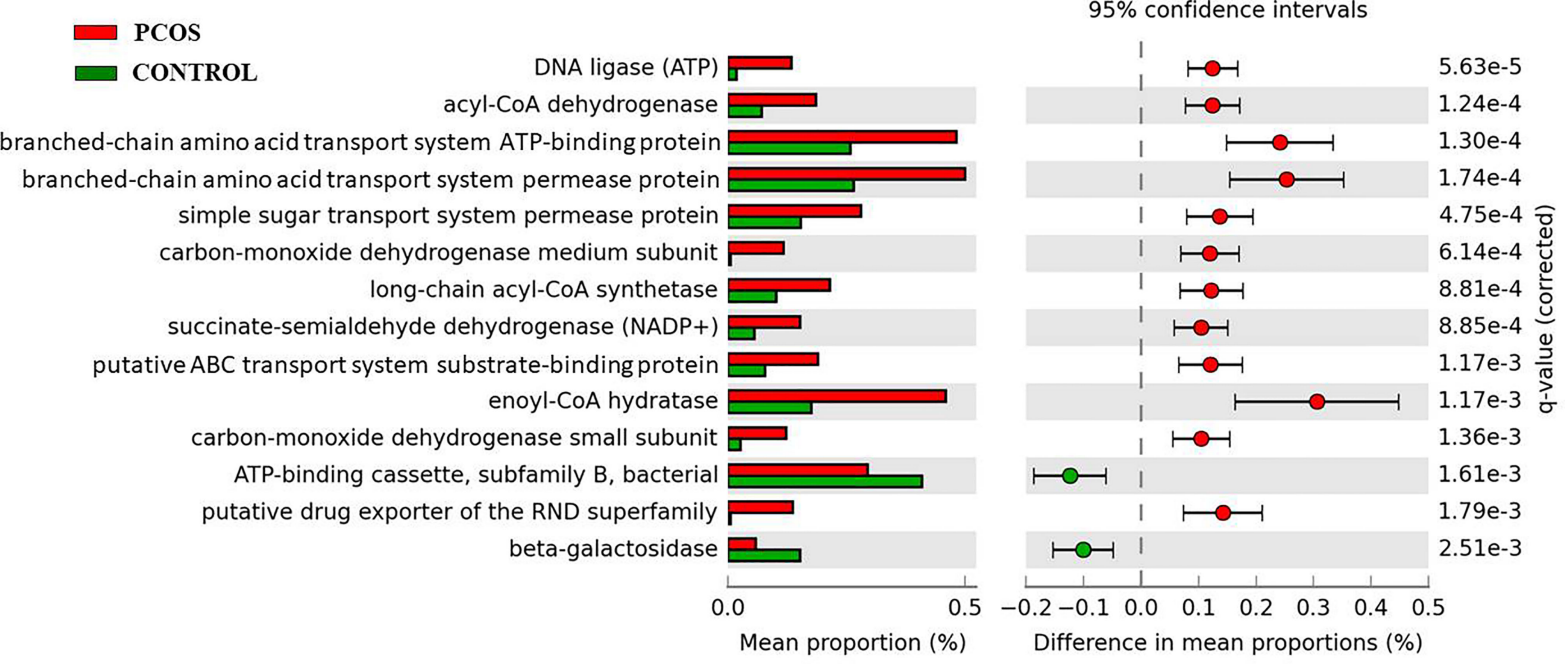
KEGG pathways analysis of PCOS and control groups.

KEGG pathways analysis by PICRUSt at genera level detected 14 main pathways with significant difference between the two groups (**Figure 4**). The PCOS group demonstrated significantly increased expressions in pathways of DNA ligase (ATP), Acyl-CoA dehydrogenase, branched-chain amino acid transport system ATP-binding protein, branched-chain amino acid transport system permease protein, simple sugar transport system permease protein, carbon-monoxide dehydrogenase medium subunit, long-chain acyl-CoA synthetase, succinate-semialdehyde dehydrogenase (NADP+), putative ABC transport system substrate-binding protein, enoyl-CoA hydratase, carbon-monoxide dehydrogenase small subunit, and putative drug exporter of the RND superfamily. Compared with the control group, the PCOS group also revealed significant decreases in beta-galactosidase and ATP-binding cassette-subfamily B-bacterial pathways.

## Discussion

Recent studies had reported the potential effect of microbiome on organs except gut. Erick et al. confirmed that the changed composition and diversity of microbiome at the tumor site might influence the immune infiltration, which might result in different prognosis after surgery of pancreatic ductal adenocarcinoma patients (24). Gopalakrishnan et al. reported the gut microbiome modulated immune response in tumor and improved the outcome of checkpoint blockade immunotherapy (25). Many studies indicated the potential effect of gut microbiome on hormonal disorders and the pathogenesis of PCOS; however, our study first measured the blood microbiome profile in PCOS compared to healthy controls.

Lower alpha diversity of gut microbiome in PCOS patients might decrease fertility, and it was associated with human obesity and metabolic disorders (26). Zeng et al. discovered a lower alpha diversity of gut microbial communities in PCOS patients with insulin resistance (4). Changed alpha diversity (19) and reduced beta diversity (27) in gut microbiome were detected between obese and non-obese PCOS patients. Our PCOS patients demonstrated a significantly decreased alpha diversity of blood microbiome compared with control participants. This was similar to most gut microbiome results (4, 16, 19). Moreover, a salivary microbiome research also revealed decreased alpha diversity in PCOS patients (20).

However, there were some different research findings. Eyupoglu et al. investigated the effect of a 3-month oral contraception administration on gut microbial composition. They detected no difference in gut microbial composition or in alpha or beta diversity between 17 overweight/obese PCOS patients and 15 age- and BMI-matched healthy controls (28). Another age- and BMI-matched cohort study indicated no significant difference in bacterial diversity of PCOS and healthy control groups (29). However, compared to women with normal glucose tolerance, pre-diabetic PCOS patients exhibited significantly lower alpha diversity and richer abundance of genus *Dorea* (29).

As for relative abundance of the blood bacteria, our linear discriminant analysis revealed significant decreases in *Bacteroidaceae* and *Ruminococcaceae* in PCOS group. Meanwhile, it was reported that gut *Ruminococcaceae* had some association with the production of short-chain fatty acid in overweight and obese PCOS participants (30), and gut *Ruminococcaceae* might stimulate production of inflammatory cytokines in type 2 diabetes patients (31). Zeng et al. also reported the association of gut *Bacteroidaceae* abundance with insulin resistance, hormonal disturbance, and inflammation markers in PCOS patients (4).

Our study discovered a significantly lower abundance of blood *Ruminococcaceae* of PCOS patients, which was consistent with some other gut microbiota studies (17, 19). Lower relative abundance of gut *Ruminococcaceae* was detected in overweight and obese participants with PCOS (30). However, Eyupoglu et al. provided evidence of an increased abundance of gut *Ruminococcaceae* in PCOS (28). Jobira et al. also confirmed a higher relative abundance of gut *Ruminococcaceae* in PCOS, which had an association with hirsutism scores (32). These deserved further study.

Our results of blood microbiome study demonstrated that the PCOS group had significantly decreased abundances in *Proteobacteria*, *Firmicutes* and *Bacteroidetes*, and a significantly richer abundance in *Actinobacteria*. Jobira et al. also discovered lower abundance of *Bacteroidetes* and higher abundance of *Actinobacteria* in gut microbiome in obese adolescents with PCOS (32). Peters et al. revealed higher abundance of some species in *Actinobacteria* phylum in obese participants compared to lean individuals (33). Thursby et al. reported that *Firmicutes* and *Bacteroidetes* might produce propionate and butyrate, modulate the short-chain fatty acids production, and even lead to dysfunctions of metabolism (34).

Our KEGG pathways analysis at genera level detected 14 main pathways with significant difference between the two groups. There were three significant increased pathways of the PCOS group that were associated with fatty acid metabolism. Acyl-CoA dehydrogenase was involved in beta-oxidation of fatty acids, enoyl-CoA hydratase also catalyzed the second step in the physiologically important beta-oxidation pathway of fatty acid metabolism (35), and long-chain acyl-CoA synthetase activated intracellular long-chain fatty acids and catalyzed fatty acids with chain lengths from 12 to 20 carbon atoms to form acyl-CoAs, which were lipid metabolic intermediates and were involved in fatty acid metabolism, various physiological processes, and membrane modifications (36).

There were some limitations deserving to improve in further study. First, our sample size was too small to make a deeper subgroup analysis of age and BMI. Second, related information about gut microbiome was not available, so we could not explore the correlation between blood and gut microbiomes between individuals or groups. Third, some other data that might influence the microbiome were missed, such as inflammatory markers, serum lipid metabolic indexes, and food habits.

## Conclusion

Our findings demonstrated that blood microbiome had a significant lower alpha diversity, different beta diversity, and significant taxonomic variations in PCOS patients compared with healthy controls.

## Data Availability Statement

The sequencing data of this study has been deposited in Sequence Read Archive (SRA) under accession number PRJNA760247. All data will be available upon request.

## Ethics Statement

The studies involving human participants were reviewed and approved by the Institutional Review Board of the First Affiliated Hospital of Xi’an Jiao Tong University (IRB No. XJTU1AF2018LSK139). The patients/participants provided their written informed consent to participate in this study.

## Author Contributions

Conception and design: QL and QingW. Development of methodology: QingW and QiW. Acquisition of data: LZ, QiW, and LiW. Analysis and interpretation of data: QingW and YB. Writing, review, and/or revision of the manuscript: QingW and KZ. Administrative, technical, or material support: LeW. Study supervision: QL.

## Funding

This work was supported by the Natural Science Basic Research Program of Shaanxi (2020JQ-952, 2018JM7073, 2017ZDJC-11), the Clinical Research Award of the First Affiliated Hospital of Xi’an Jiaotong University, China (XJTU1AF-2018-017, XJTU1AF-CRF-2019-002), the Key Research and Development Program of Shaanxi (2017ZDXM-SF-068, 2019QYPY-138), the Innovation Capability Support Program of Shaanxi (2017XT-026, 2018XT-002), and the Medical Research Project of Xi’an Social Development Guidance Plan (2017117SF/YX011-3). The funders had no role in study design, data collection and analysis, decision to publish, or preparation of the manuscript.

## Conflict of Interest

The authors declare that the research was conducted in the absence of any commercial or financial relationships that could be construed as a potential conflict of interest.

## Publisher’s Note

All claims expressed in this article are solely those of the authors and do not necessarily represent those of their affiliated organizations, or those of the publisher, the editors and the reviewers. Any product that may be evaluated in this article, or claim that may be made by its manufacturer, is not guaranteed or endorsed by the publisher.
