# Supplementary figures and images for: Blood Bacterial 16S rRNA Gene Alterations in Women With Polycystic Ovary Syndrome
Source: Front Endocrinol (Lausanne). 2022 Feb 24;13:814520. doi: 10.3389/fendo.2022.814520 (PMC8908962; doi:10.3389/fendo.2022.814520)

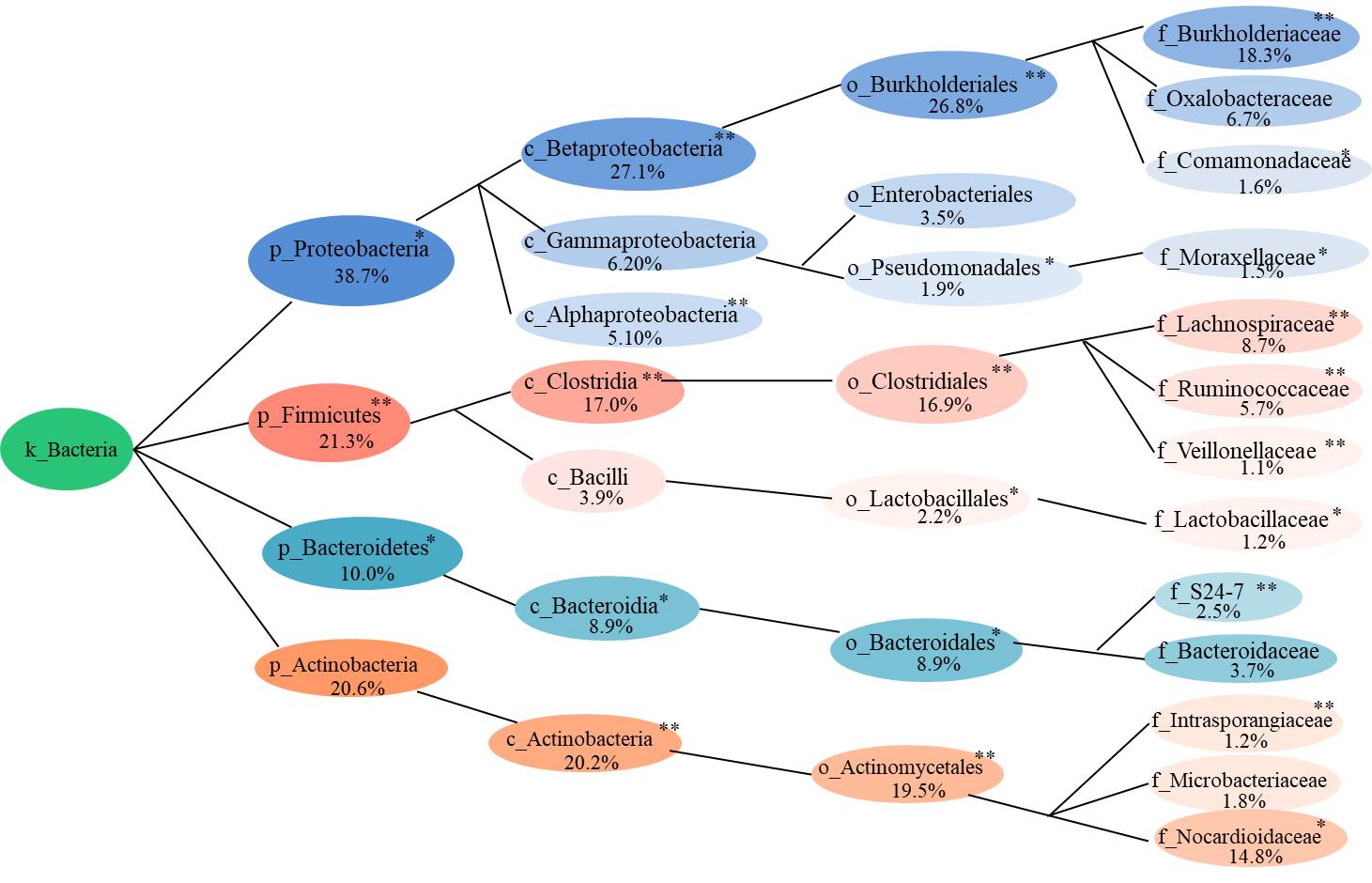

Supplement: Supplementary file 1 [file Image_1.jpeg]

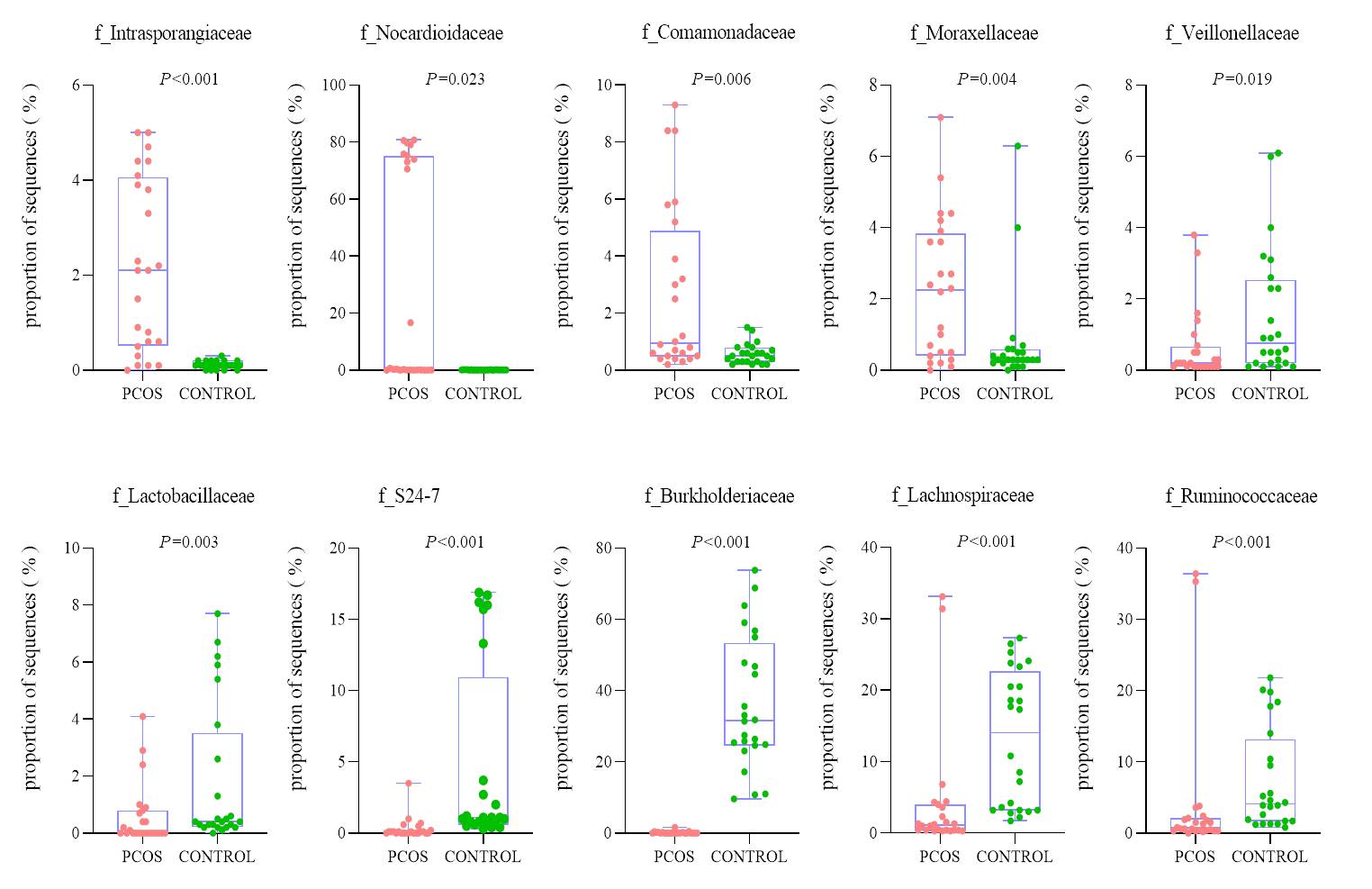

Supplement: Supplementary file 2 [file Image_2.jpeg]
